# Supplementary material for: Quantitative and qualitative evaluation of a learning model based on workstation activities
Source: PLoS One. 2020 Aug 5;15(8):e0236940. doi: 10.1371/journal.pone.0236940 (PMC7406050; doi:10.1371/journal.pone.0236940)
Supplement: Attachment — Submitted filename: Rebuttal Letter-PlosOne.docx [file pone.0236940.s002.docx]

**Reviewers' comments:**

1. Is the manuscript technically sound, and do the data support the conclusions?

The manuscript must describe a technically sound piece of scientific research with data that supports the conclusions. Experiments must have been conducted rigorously, with appropriate controls, replication, and sample sizes. The conclusions must be drawn appropriately based on the data presented.

Reviewer #1: No

We have improved reporting and clarified details to satisfy reviewer.

2. Has the statistical analysis been performed appropriately and rigorously?

Reviewer #1: Yes

Thanks.

3. Have the authors made all data underlying the findings in their manuscript fully available?

The [PLOS Data policy](http://secure-web.cisco.com/1xihvk-7GDCsyfkXXe9dd0SS1ZfiOeN4WzpuSsvhgpN4T_X4_OpF60Lk47DeK2hu-6OB0QKUMChtyHnw-tZ4KvDhMYxg0byEA2AtUTnsV8vXRpGyEWrZibL73uca9IDn8AqPmUqj4HFJeyMN62Rt5e-LbWMqw57X0K0OGIqhYqBwBdox584XctP2fbAtpzAh_sx5603PX-nsNPCrWlWgKarVaCWJB4EheML9HrN4GGYZ4iKGeR2wRNfvFHoh4dDj-cN43yGprlvX5Gi0BEsCsHtJkEkC9J_5rer_sIH5h0xo-okALLUmWkg2e2oyJcYkEC6vgIsP39apAOARx7CBh4Q/http%3A%2F%2Fwww.plosone.org%2Fstatic%2Fpolicies.action%23sharing) requires authors to make all data underlying the findings described in their manuscript fully available without restriction, with rare exception (please refer to the Data Availability Statement in the manuscript PDF file). The data should be provided as part of the manuscript or its supporting information, or deposited to a public repository. For example, in addition to summary statistics, the data points behind means, medians and variance measures should be available. If there are restrictions on publicly sharing data—e.g. participant privacy or use of data from a third party—those must be specified.

Reviewer #1: Yes

Thanks. We add full anonymized data as supplementary material.

4. Is the manuscript presented in an intelligible fashion and written in standard English?

PLOS ONE does not copyedit accepted manuscripts, so the language in submitted articles must be clear, correct, and unambiguous. Any typographical or grammatical errors should be corrected at revision, so please note any specific errors here.

Reviewer #1: Yes

Thanks.

5. Review Comments to the Author

Reviewer #1: The authors describe a learning model that by modern educational research standards would be obviously better than pure lecture and the benefit would show up to a greater extent on more conceptually difficult to answer questions, unless technical details were poorly done. There is not an open research question to answer here. This is a quality assurance project (were these materials well designed at the details level).

We apologize our research question was not clearly posed in the previous version.

Please note we introduced the WSLA in 2016, as a flexible learning instrument in our university. Up to that moment, most teaching followed classical methodology and basic and clinical sciences were poorly integrated. Thus, our main motivation with this work was to evaluate quantitative and qualitatively whether and how WSLA affects academic performance and to understand student´s experience with the methodology. We found WSLA useful to integrate learning at the higher cognitive level while promoting student engagement. We have revised the manuscript to make our question clearer. See Abstract (first paragraph) and Introduction (last paragraph).

While Bloom's Taxonomy remains popular in applied contexts, it is terribly outdated. The full taxonomy has many levels beyond just higher and lower as a binary. There never was a clear empirical basis for the original taxonomy, nor has there been a systematic validation of it or the revised one. "Apply" is a hopelessly broad category. Webb's Domains of Knowledge could be used, since it actually has some empirical support. If one wants to use a general framework from this century, I would suggest Chi and colleagues' ICAP framework. Easy and clear in how to use it in research; strong empirical validation.

We thank the reviewer for bringing our attention to this very important point. We feel that in the context of our study, Bloom’s and Webb’s definitions apply in relation to learning goals and assessment. The empirical character of the learning framework is another aspect to consider. We appreciate the reviewer suggestions to rely on the ICAP framework, but because our practice was focused on learning objectives, our study adopted Bloom´s taxonomy. Notably, we don’t see Bloom´s taxonomy as binary. The fact that we identified difference at low and high levels rather reflects the effect in the continuum of the cognitive spectrum.

However, we feel that incorporating the reviewer´s perspectives benefits the analysis of our work. Indeed, the WSLA as a flexible learning instrument allows combining multiple perspectives. Thus, we have substantially edited the Introduction to connect the evolution from Bloom’s and Webb’s considerations into more constructive and empirically demonstrated frameworks, such as ICAP. Later, at the Discussion, we revolve around these ideas to extend the reach of our conclusion further and we suggest indeed that adopting ICAP is desirable for a future work. We feel the manuscript has gained a wider view. See Introduction and Discussion.

As an empirical study, there were also many problems. The two cohorts were not checked for equivalence on incoming standards.

We appreciate this comment. We have added information on incoming standards in the revised version. See page 5.

Focusing on just one model is thin from a generalizability standpoint.

We understand the reviewer meant the WSLA model. Please, note that our motivation was to assess this model versus the traditional. Of course, we are running different programs and models in our everyday health science education. Those are now under review in other journals. We feel it is important for clarity sake to address them one by one.

Using one multiple choice question is terrible from a measurement perspective.

Indeed, we had many multiple choice questions to measure each learning objective. However, we included only those questions meeting statistical criteria regarding similar difficulty and discrimination index and quality of questions in the two cohorts to avoid confounding factors. While this was specified in Materials and Methods of the previous version, we have revised the current version to make even clearer. See page 6.

The interview analysis had a clear bias of interpretation: when the students asserted that the combination of lecture and workstation was important, the authors assumed (ironically!) that the students were wrong and their answers reflected cultural bias.

We fully agree. Thank you for highlighting this. This was removed in the masnucript accordingly.
